# Supplementary material for: Comparative transcriptome profiling suggests the role of phytohormones in leaf stalk-stem angle in melon (Cucumis melo L.)
Source: PeerJ. 2024 Nov 18;12:e18467. doi: 10.7717/peerj.18467 (PMC11580662; doi:10.7717/peerj.18467)
Supplement: Supplemental Information 1 [file peerj-12-18467-s001.docx]

Supplementary Material

Comparative transcriptome profiling suggests the role of phytohormones in leaf stalk-stem angle in melon (*Cucumis melo* L.)

Jian-Cai Mao^†^, Hao-Jie Wang^†^, Jun-Hua Li, Jun-Yan Yang, Yong-Bing Zhang* and Hai-Bo Wu*

*** Correspondence:** wuhaibo@xaas.ac.cn; hamimelon@xaas.ac.cn

# Supplementary Figures and Tables

## Supplementary Tables

**Supplementary Table 1** Information on phytohormone standardized products

| **Name** | **Batch number** | **Purity** | **Abbreviation** | **CAS** | **manufacturers** |
| --- | --- | --- | --- | --- | --- |
| Gibberellin A4 | 2597-019A10 | 95.2% | GA4 | 468-44-0 | TLC |
| Gibberellin A5 | 15-MP-73-4 | 95.0% | GA5 | 561-56-8 | TLC |
| Gibberellin A7 | 2668-042A6 | 99.6% | GA7 | 510-75-8 | TLC |
| Gibberellin A8 | 3-HAD-62-1 | 95.0% | GA8 | 7044-72-6 | TRC |
| Gibberellin A19 | GA19-B | 98.0% | GA19 | 6980-44-5 | olchemim |
| Gibberellin A24 | GA24-A | 95.0% | GA24 | 19427-32-8 | olchemim |
| Gibberellin A51 | GA51-B | 95.0% | GA51 | 56978-14-4 | olchemim |
| Abscisic acid | 21B090-A2 | 99.5% | ABA | 14375-45-2 | ZZSTANDARD |
| Salicylic acid | P1998072 | 99.0% | SA | 69-72-7 | Sigma |
| salicylic acid  2-O-β-D- glucose | 2-RGP-104-1 | 95.0% | SAG | 10366-91-3 | TRC |
| Jasmonate acid | 14-SBL-87-1 | 97.0% | JA | 3572-66-5 | TRC |
| 3-Indoleformic acid | P1998119 | 98.0% | ICA | 771-50-6 | Sigma |
| Indole-3-acetic acid | H1521055 | 97.5% | IAA | 87-51-4 | CNPGC |
| 3-Indolebutyric acid | E1929056 | 98.0% | IBA | 133-32-4 | Aladdin |
| Cis-zeatin | 2952-026A3 | 97.8% | CZ | 32771-64-5 | TLC |
| Trans-zeatin riboside | E1930262 | 97.0% | TZR | 6025-53-2 | Aladdin |
| N6-(delta 2-Isopentenyl)-adenine | BCCC9151 | 99.0% | IP | 2365-40-4 | Sigma |
| N6-isopentenyladen osine-D6 | E2125196 | 98.0% | IPA | 7724-76-7 | Aladdin |
| DL-dihydrozeatin | 2877-040A2 | 99.6% | DZ | 14894-18-9 | TLC |
| Benzyladenine | I1925201 | 99.0% | 6-BA | 1214-39-7 | Aladdin |
| 1-Aminocyclopropane-1-carboxylic acid | K1520059 | 97.5% | ACC | 22059-21-8 | Aladdin |
| Dihydrojasmonic acid | 21J142-D4 | 98.0% | DHJA | 3572-64-3 | ZZSTANDARD |
| N-[(-)-jasmonoyl]-(S )-isoleucine | MR200922001 | 98.5% | JA-IIe | 28838-58-6 | SCRBIO |
| Methyl 2-(1H-indol-3-yl)acetate | P1998125 | 99.0% | Me-IAA | 1912-33-0 | Sigma |
| Indole-3-carboxaldehyde | G2021117 | 97.0% | ICAId | 487-89-8 | Aladdin |
| Trans-Zeatin | Z0876 | 97.0% | TZ | 1637-39-4 | Sigma |
| Methyl jasmonate | D2109301 | 98.0% | MEJA | 39924-52-2 | Aladdin |
| Brassinolide | J20J11H18372 | 96.8% | BR | 72962-43-7 | yuanye |
| Gibberellic acid A3 | D1823038 | 95.0% | GA3 | 77-06-5 | Aladdin |
| Melatonin | E2013132 | 97.5% | MT | 73-31-4 | Aladdin |
| Kinetin | I2025068 | 99.0% | Kt | 525-79-1 | Aladdin |
| 12-oxophytodienoic acid | E665-B | 95.0% | OPDA | 80736-41-0 | SCRBIO |
| GR24 | HR1192W3 | 98.0% | GR24 | 76974-79-3 | CHENGUANG |

**Supplementary Table 2** The differential gene screening results and summary information are as follows

| Sample | Reads No. | Base(bp) | Clean reads(100%) | Q30(bp) | N(%) | Q20(%) | 30(%) |
| --- | --- | --- | --- | --- | --- | --- | --- |
| Y164-1 | 46339280 | 6997231280 | 97.78 | 6640711333 | 0.010030 | 97.11 | 94.90 |
| Y164-2 | 39939290 | 6030832790 | 97.82 | 5725941333 | 0.010030 | 97.13 | 94.94 |
| Y164-3 | 42237268 | 6377827468 | 97.81 | 6048207932 | 0.009973 | 97.07 | 94.83 |
| Z151_1 | 43031800 | 6497801800 | 97.91 | 6169363419 | 0.009953 | 97.13 | 94.95 |
| Z151_2 | 41031136 | 6195701536 | 97.87 | 5877528351 | 0.009944 | 97.09 | 94.86 |
| RZ151_3 | 37617376 | 5680223776 | 97.73 | 5373773078 | 0.009923 | 96.94 | 94.60 |

**Supplementary Table 3** Details of alingment sequences

| Sample | Clean_Reads | Total_Mapped | Multiple_Mapped | Uniquely_Mapped |
| --- | --- | --- | --- | --- |
| Y164-1 | 45312670 | 44391868 (97.97%) | 911378 (2.05%) | 43480490 (97.95%) |
| Y164-2 | 39069918 | 30911939 (79.12%) | 602588 (1.95%) | 30309351 (98.05%) |
| Y164-3 | 41311842 | 40011004 (96.85%) | 792338 (1.98%) | 39218666 (98.02%) |
| Z151_1 | 42133220 | 41332856 (98.10%) | 858362 (2.08%) | 40474494 (97.92%) |
| Z151_2 | 40158542 | 28495631 (70.96%) | 558209 (1.96%) | 27937422 (98.04%) |
| RZ151_3 | 36764984 | 35928750 (97.73%) | 756678 (2.11%) | 35172072 (97.89%) |

**Supplementary Table 4** Quantitative ion pairs for analysis

| **No.** | **Abbr.** | **Ionization mode.** | **Q1** | **Q3** | **DP** | **CE** | **CXP** |
| --- | --- | --- | --- | --- | --- | --- | --- |
| 1 | GA1 | ESI(-) | 347.1 | 273.2 | -108 | -32 | -22 |
| 2 | GA4 | ESI(-) | 330.9 | 243.1 | -85 | -26 | -29 |
| 3 | GA5 | ESI(-) | 329.0 | 145.0 | -63 | -30 | -11 |
| 4 | GA7 | ESI(-) | 329.1 | 223.1 | -69 | -25 | -20 |
| 5 | GA8 | ESI(-) | 363.0 | 275.0 | -92 | -20 | -11 |
| 6 | GA19 | ESI(-) | 361.3 | 177.1 | -84 | -41 | -11 |
| 7 | GA24 | ESI(-) | 345.0 | 257.0 | -75 | -20 | -25 |
| 8 | GA51 | ESI(-) | 331.0 | 243.0 | -64 | -35 | -11 |
| 9 | ABA | ESI(-) | 263.1 | 153.2 | -60 | -18 | -8 |
| 10 | SA | ESI(-) | 136.8 | 93.0 | -31 | -22 | -3 |
| 11 | SAG | ESI(-) | 299.0 | 136.9 | -50 | -18 | -11 |
| 12 | JA | ESI(-) | 209.0 | 59.0 | -33 | -16 | -20 |
| 13 | ICA | ESI(-) | 160.1 | 116.0 | -35 | -20 | -11 |
| 14 | IAA | ESI(+) | 176.0 | 130.1 | 50 | 23 | 12 |
| 15 | IBA | ESI(+) | 204.1 | 130.0 | 13 | 29 | 16 |
| 16 | CZ | ESI(+) | 220.2 | 136.0 | 39 | 23 | 16 |
| 17 | TZR | ESI(+) | 352.0 | 219.9 | 34 | 28 | 10 |
| 18 | IP | ESI(+) | 204.2 | 136.1 | 36 | 22 | 7 |
| 19 | IPA | ESI(+) | 336.1 | 204.1 | 47 | 26 | 11 |
| 20 | DZ | ESI(+) | 222.2 | 136.1 | 34 | 28 | 16 |
| 21 | 6-BA | ESI(+) | 226.1 | 91.0 | 60 | 30 | 11 |
| 22 | ACC | ESI(+) | 102.0 | 56.0 | 10 | 20 | 15 |
| 23 | DHJA | ESI(-) | 211.0 | 59.0 | -61 | -18 | -8 |
| 24 | JA-IIE | ESI(-) | 322.0 | 130.0 | -50 | -30 | -11 |
| 25 | ME-IAA | ESI(+) | 190.0 | 130.0 | 38 | 27 | 9 |
| 26 | ICAId | ESI(-) | 144.1 | 115.2 | -70 | -37 | -12 |
| 27 | TZ | ESI(+) | 220.1 | 136.0 | 46 | 25 | 19 |
| 28 | MEJA | ESI(+) | 225.1 | 150.9 | 32 | 21 | 12 |
| 29 | BR | ESI(+) | 481.3 | 445.4 | 46 | 15 | 14 |
| 30 | GA3 | ESI(-) | 345.2 | 143.0 | -63 | -35 | -11 |
| 31 | Mt | ESI(+) | 233.2 | 174.1 | 44 | 23 | 8 |
| 32 | Kt | ESI(+) | 216.2 | 81.0 | 27 | 29 | 9 |
| 33 | Gr24 | ESI(+) | 299.2 | 185.1 | 33 | 14 | 22 |
| 34 | PPDA | ESI(-) | 291.2 | 165.1 | -85 | -26 | -19 |

No.: number; Abbr.: compound name abbreviation; Q1: parent ion; Q3: daughter ion; CE: collision voltage; DP: de-clustering voltage; CXP: collision chamber ejection voltage.

## Supplementary Figures


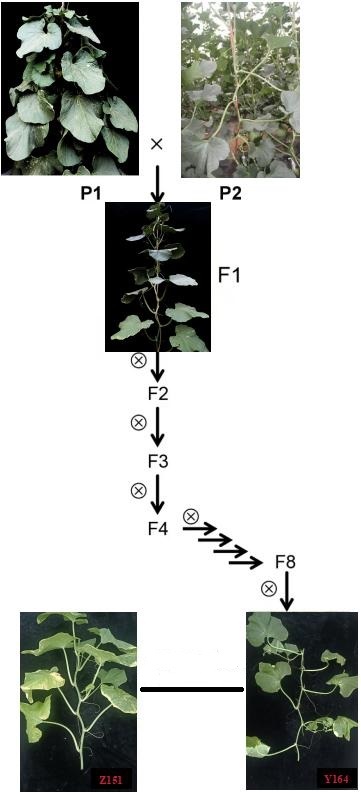


**Supplementary Figure S1** The process of generating the mutant Z151. P_1_ is a dwarf melon and P_2_ is a normal height melon, and the F_1_ of the cross between the two showed normal plant height, and 120 stable populations of recombinant inbred lines were obtained after single-particle transmission to the F_8_ generation. Z151 and Y164 are two lines from the RIL population and were observed to be identical in all other traits except leaf stalk-stem angle.
